# Discourse abilities in euthymic elderly patients with bipolar disorder: a preliminary study

**DOI:** 10.1590/1980-5764-DN-2022-0067

**Published:** 2023-05-05

**Authors:** Maria Gabriela Valeriano, Renné Alegria, Orestes Vicente Forlenza, Marcia Radanovic

**Affiliations:** 1 Universidade de São Paulo, Faculdade de Medicina, Departamento de Psiquiatria, Laboratório de Neurociências, São Paulo SP, Brazil.

**Keywords:** Language, Bipolar Disorder, Aged, Narration, Cognition, Idioma, Transtorno Bipolar, Idoso, Narração, Cognição

## Abstract

Cognitive impairment has been well described in euthymic patients with bipolar disorder (BD), as well as in elderly patients. Language disturbances are less studied, and several inconsistencies are reported in the literature. Most language studies focus on verbal fluency and semantic alterations, with a lack of studies addressing discursive abilities in BD.

**Objective:**

The aim of this study was to evaluate discourse abilities in euthymic elderly individuals with BD.

**Methods:**

We studied 19 euthymic elderly patients with BD and a control group of non-BD, which performed a cognitive assessment of attention, memory, executive functions, and visual abilities. All participants produced a description from the Cookie Theft Picture in oral and written modalities that was analyzed according to micro- and macrolinguistic aspects. Generalized linear models were performed to compare intergroup linguistic performance and to determine whether any cognitive domain was associated with linguistic outcomes.

**Results:**

The BD group produced more cohesion errors in the oral and written modalities (p=0.016 and p=0.011, respectively) and fewer thematic units in the oral modality (p=0.027) than the control group.

**Conclusions:**

BD patients presented minimal changes in the descriptive discourse task. The BD group produced more cohesion errors than the control group in the oral (p=0.016) and written discourse (p=0.011); also, the BD group produced fewer thematic units than controls in the oral discourse (p=0.027).

## RESUMO

Déficits cognitivos têm sido descritos em pacientes com transtorno bipolar (TB) em fase eutímica, bem como em idosos. Alterações linguísticas são menos estudadas, e os achados de literatura são inconsistentes. A maioria dos estudos em linguagem baseia-se em avaliações de fluência verbal e alterações semânticas, havendo escassez de trabalhos que abordem as habilidades discursivas no TB.

**Objetivo:**

Avaliar as habilidades discursivas em indivíduos idosos eutímicos com TB.

**Métodos:**

Estudamos 19 pacientes idosos eutímicos com TB e um grupo de idosos sem TB e cognitivamente saudáveis, que realizaram avaliação cognitiva da atenção, memória, funções executivas e habilidades visuoespaciais. Todos os participantes produziram uma descrição da Prancha do Roubo dos Biscoitos nas modalidades oral e escrita, que foram analisadas de acordo com aspectos micro e macrolinguísticos. Análises por meio de modelos lineares generalizados foram realizados para comparar o desempenho linguístico entre os grupos e para determinar se algum domínio cognitivo estava associado a esse desempenho.

**Resultados:**

O grupo TB produziu mais erros de coesão nas modalidades oral e escrita (p=0,016 e p=0,011, respectivamente) e menos unidades temáticas na modalidade oral (p=0,027) do que o grupo controle.

**Conclusão:**

Os pacientes com TB apresentaram alterações leves na tarefa discursiva. O grupo TB produziu maior número de erros de coesão do que o grupo controle no discurso oral (p=0,016) e escrito (p=0,011). Além disso, o grupo TB produziu menor número de unidades temáticas do que os controles na tarefa de discurso oral (p=0,027).

## INTRODUCTION

Bipolar disorder (BD) is a chronic mental condition characterized by the oscillation between recurrent periods of elevated mood (manic, hypomanic, or mixed episodes) that alternate with periods of depression (depressive episodes), interspersed with periods of absence of affective symptoms and apparent clinical recovery, known as euthymia. BD in geriatric patients has two presentations: the disease that first manifests in this age group (late-onset BD, usually presenting milder symptoms) and the disease that begins before senescence and persists throughout life. In elderly patients with BD, manic episodes seem to be less severe, with fewer symptoms of hypersexuality, impulsivity, and a predominance of mood elation and loss of insight. Mixed episodes (in which manic and depressive symptoms co-occur) are more frequent in elderly patients with early-onset BD^1^.

We are currently experiencing the emergence of a group of aging people with BD who require specific studies focused on their condition, given that cognitive and functional impairments are common features of the long-term outcome in older age. Cognitive deficits in BD are common and include impairment in episodic memory, executive functions, attention, and processing speed^2^. This impairment may persist in euthymia and is critically associated with lower quality of life, occupational outcomes, and, ultimately, functional impairment in late-life BD. Verbal memory and executive functions are the most frequently affected cognitive domains in this subgroup of patients^3^. Factors associated with the risk of cognitive decline and subsequent dementia in BD include the number of affective (especially manic) episodes, duration of illness, presence of psychotic manifestations, and morphological changes of brain structures (enlargement of the lateral ventricles, deep white matter hyperintensities, and gray matter reduction in the inferior frontal gyri of the dorsolateral prefrontal cortices)^4^.

Given the heterogeneity of this involvement among patients, there is no consensus about which mechanism (or combinations) is responsible for cognitive impairment in BD: deficient brain reserve, allostatic load (associated with the secretion of neuroinflammatory substances), and increased cerebral vascular burden. Furthermore, the effects of chronic intake of medications, residual mood symptoms, and clinical comorbidities may also be involved in this process^5^.

Language and speech abnormalities are known to occur in BD patients. During manic episodes, pressure of speech, with increased rapidity of speech and racing thoughts, as well as rapidly shifting between discourse structures, increased verbosity, and clang (i.e., phonetic) associations, are frequently observed. In depressive episodes, poverty of speech and increased pause times are the most common findings^6^. However, fewer studies have addressed language impairment in euthymic patients, and most have focused on analyzing specific tasks, such as verbal fluency and naming, embedded in more comprehensive neuropsychological testing^7^. Alterations in semantic processing, impaired verbal associations, abnormal prosody, and decreased verbal fluency are among the linguistic deficits already reported in the literature^8^. Clustering analysis in verbal fluency tasks shows that patients with BD are prone to produce less coherent category clusters than cognitively healthy controls^9^. Such disorders of linguistic processing have been confirmed by neuroimaging studies^10^.

Currently, increased value has been placed on language evaluation based in more ecological settings, such as spontaneous speech and discourse production, which may prove more useful to detect those alterations that might produce a substantial functional impact in routine activities when compared to metalinguistic processes as those employed in language tests. To the best of our knowledge, only one study examined discourse production in BD from a neurolinguistic perspective^11^. Our objective was to study the oral and written discourse abilities in a group of euthymic elderly patients with BD.

## METHODS

We studied 19 bipolar patients aged 60 years and above, with BD type I or II, and euthymic at the evaluation time. The control group was composed of 19 cognitively healthy elderly patients recruited from the same psychogeriatric unit. Inclusion criteria for bipolar patients were as follows: diagnosis of BD type I or II according to the *ICD-10* criteria^12^, euthymia defined as a maximum score of 7 in the 21-item Hamilton Rating Scale for Depression (HRSD)^13^, and 4 in the Young Mania Rating Scale (YMRS)^14^ throughout the preceding month. Subjects aged 60 years and older were recruited as controls and met the criteria established by the Mayo Older American Normative Studies for the diagnosis of normal cognition for individuals aged 55 years and above^15^. Exclusion criteria for both groups were as follows: history or clinical evidence (including neuroimaging) of medical, neurological, or psychiatric illness (other than BD) that could influence cognitive performance; history of alcohol or drug abuse; auditory, visual, or motor impairment that might preclude cognitive testing. All participants were submitted to a cognitive evaluation assessing attention, working memory, executive functions, episodic (verbal and nonverbal) memory, and visual abilities.

In all, 17 patients had BD type I, and 2 had BD type II. Ten patients received lithium therapy (daily doses ranging from 300 to 1200 mg). Six patients were prescribed two mood-stabilizing agents (lithium and lamotrigine). This study was approved by the local research ethics committee and performed following the Declaration of Helsinki as revised in 1989. All participants gave their informed consent before enrollment in the study.

### Language assessment

Patients and controls produced a discourse in oral and written modalities based on the Cookie Theft Picture^16^. All participants performed the oral description followed by the cognitive tests, and after 30–40 min, they were instructed to look again at the picture and produce a written text. Oral descriptions were recorded and transcribed verbatim for posterior analysis. There was no time limit for responses.

### Discourse analysis

Text structure was analyzed at the micro- (sentence) and macrolinguistic (discourse) levels according to the criteria proposed by Perlini et al.^11^ (for details, see Supplementary material).

### Statistical analysis

Intergroup performance was compared using a two-way analysis of variance (ANOVA) with repeated measures with group as a between-subject factor and narrative as a within-subject factor for each group on 13 criteria: words, utterances, mean length of utterances, paragrammatic errors (%), omissions (%), paraphasias (%), syntactic completeness (%), sentence complexity (%), global coherence errors (%), local coherence errors (%), cohesion errors (%), lexical informativeness (%), and thematic units. Intragroup differences in narrative structure (oral vs. written) were assessed through a one-way ANOVA group*modality as a fixed factor and the 13 linguistic measures as dependent variables. A hierarchical linear model regression was used to investigate whether any cognitive measure could predict those linguistic variables altered in the BD group (% of cohesion errors and number of thematic units). Age and schooling were added as covariates for all analyses. A p-value of <0.05 was set for all analyses.

## RESULTS

Demographic, clinical, and cognitive data of the sample are displayed in Table 1. BD patients performed poorer than controls in animal and phonemic fluencies, in the TMT-A test, and in the TROG-2, which denote impairment in attentional/executive abilities and syntactic processing.

**Table 1:** Demographic, clinical, and cognitive data of the sample*.

Variable		Control	BD	p (bi-caudal)
Age		72.4 (3.8)	68.0 (4.3)	0.003
Schooling		13.1 (3.7)	11.1 (4.7)	0.148
Sex†				
	Female	15	13	0.920
	Male	5	6	
HRSD		0.6 (1.3)	5.6 (2.3)	<0.0001
YMRS		NA	2 (1.9)	NA
TMT (A), s		42.6 (10.8)	79.2 (38.6)	0.001
Digit span				
	Forward	5.5 (0.9)	5.5 (0.8)	0.922
	Backward	3.8 (0.9)	3.5 (1.2)	0.414
SKT				
	Naming objects	12.0 (0)	11.9 (0.2)	0.873
	Immediate recall	6.5 (1.8)	5.6 (1.8)	0.121
	Delayed recall	7.5 (1.9)	7.0 (1.7)	0.362
	Recognition	11.5 (0.8)	11.3 (1.0)	0.534
VF animals		17.5 (3.1)	13.9 (4.9)	0.015
FAS-COWA		40.3 (10.1)	27.2 (12.7)	0.003
TROG-2		94.9 (7.2)	83.6 (14.9)	0.006

Notes:

Abbreviations: BD: bipolar disorder; HRSD: Hamilton Rating Scale for Depression; YMRS: Young Mania Rating Scale; TMT: Trail Making Test; SKT: Short Cognitive Test; VF: verbal fluency; FAS-COWA: FAS Category Oral Word Association; TROG-2: Test for the Reception of Grammar-2; NA: not applicable.

* Intergroup-related differences in demographic and clinical data were assessed through Student’s t-test with Bonferroni’s correction for multiple comparisons.

† Pearson’s chi-square test.

Table 2 depicts the individuals’ performance in oral and written narrative production. Intergroup analyses for the oral narrative task showed that the BD group produced more cohesion errors, F(1,25)=6.667; p=0.016; η^2^p=0.211, and fewer thematic units, F(1,34)=5.522; p=0.027; η^2^p=0.181. The BD group produced more cohesion errors in the written discourse task than controls, F(1,34)=7.159; p=0.011; η^2^p=0.174.

**Table 2:** Linguistic performance of individuals in oral and written discourse production.

Variable			Controls	BD	F	p (bi-caudal)	η^2^p
Microlinguistic analysis							
	Words	Oral	115.1 (61.5)	92.3 (39.6)	0.173	0.681	0.007
		Written	82.3 (36.4)	58.4 (32.2)	0.261	0.613	0.008
	Utterances	Oral	19.0 (8.4)	14.2 (5.6)	0.943	0.341	0.036
		Written	10.9 (4.4)	7.0 (3.7)	2.782	0.105	0.076
	Mean length of utterance	Oral	5.8 (0.9)	6.4 (0.7)	3.499	0.073	0.123
		Written	7.5 (1.8)	8.5 (2.4)	3.232	0.081	0.087
	Paragrammatic errors (%)	Oral	0.1 (0.2)	0.9 (1.3)	0.157	0.695	0.006
		Written	0.1 (0.3)	1.7 (3.7)	0.223	0.640	0.007
	Omissions (%)	Oral	1.9 (3.2)	0.6 (1.7)	3.929	0.059	0.136
		Written	5.6 (8.8)	7.2 (10.9)	0.900	0.349	0.026
	Paraphasias (%)	Oral	0.0 (0.0)	0.0 (0.3)	0.529	0.474	0.021
		Written	0.0 (0.0)	0.6 (1.9)	0.045	0.834	0.001
	Sentence completeness (%)	Oral	64.3 (15.6)	67.7 (19.4)	0.084	0.774	0.003
		Written	80.5 (23.5)	77.8 (18.3)	0.001	0.979	0.000
	Sentence complexity (%)	Oral	16.2 (9.8)	13.3 (9.2)	1.801	0.192	0.067
		Written	27.3 (14.8)	29.5 (19.3)	0.778	0.384	0.022
Macrolinguistic analysis							
	Global coherence errors (%)	Oral	19.4 (11.0)	20.6 (17.5)	6.667	0.016	0.211
		Written	4.5 (9.4)	7.9 (12.8)	2.113	0.155	0.059
	Local coherence errors (%)	Oral	2.3 (3.1)	2.4 (3.5)	5.522	0.027	0.181
		Written	2.5 (8.2)	4.9 (12.5)	7.159	0.011	0.174
	Cohesion errors (%)	Oral	1.9 (3.5)	3.7 (3.9)	0.527	0.475	0.021
		Written	1.1 (3.3)	7.5 (10.8)	0.048	0.828	0.001
	Lexical informativeness (%)	Oral	75.6 (13.8)	66.3 (20.7)	0.009	0.925	0.000
		Written	93.0 (11.8)	88.9 (16.3)	0.463	0.501	0.013
	Thematic units	Oral	7.6 (0.5)	6.2 (2.0)	2.654	0.116	0.096
		Written	7.5 (0.6)	6.5 (1.6)	1.103	0.301	0.031

Abbreviations: BD: bipolar disorder; F: statistics.

Intragroup comparison between narrative structures (oral vs. written) showed that both modalities were similar in syntactic completeness, local coherence, cohesion, percentage of paragrammatic errors and paraphasias, the number of thematic units (all p>0.05), oral narratives had more words and utterances (p<0.01), and less lexical informativeness (p<0.01) in controls and BD patients. Intragroup dissociations included longer utterances and increased syntactic complexity in the written narrative in BD; fewer global coherence errors in the written discourse for the control group. Multiple linear regression analysis did not show any association between the number of thematic units and cohesion errors with any cognitive measure in the BD group.

## DISCUSSION

Language studies in individuals with BD have long focused on the language production of patients in manic and depressive states^17^ or the contrast between BD and schizophrenia^18^. More recently, interest has turned to the cognitive performance of euthymic bipolar patients, and several studies have demonstrated impairment in executive functions, working and episodic memory, attention, and processing speed. However, in most studies, language functions are poorly examined, considering that tests of verbal fluency and verbal memory, which are the most studied, do not address the functional aspects of language use. Language skills are better evaluated through tests that include their phonological, morphosyntactic, lexical-semantic, and discourse elements.

We aimed at studying the discourse abilities of a cohort of elderly patients with euthymic bipolar in the oral and written modalities, considering that discourse abilities may better represent the natural use of language, albeit not as much as spontaneous speech. In a preliminary study on cognitive performance in euthymic elderly patients with BD, we found that language was the most effective domain for differentiating BD from controls^19^. In a study using a more comprehensive language battery^20^, the authors described language alterations in elderly patients with euthymic bipolar compared to cognitively healthy elderly patients, especially in language expression tasks (object description, naming, and concept definition), suggestive of semantic network impairment. We were able to find two additional studies focusing on semantic processing in BD that report decreased semantic priming effect^21^ and impaired semantic inhibition^22^. Syntactic processing alterations were also reported in the literature^11,20^ that demonstrated grammatical processing deficits in BD patients compared to controls. Such findings were corroborated by electrophysiological measures^23^.

Therefore, we concluded that the next natural step would be to address the discourse abilities of elderly patients with BD. Discourse abilities are more representative of “real-world” language and correspond to individuals’ degree of communicative competence because they are a spontaneous production, even if constrained by a predetermined stimulus. We chose to analyze the descriptive discourse from the Cookie Theft Picture in oral and written modalities. Picture description tasks allow the identification of complex cognitive-linguistic impairments through the analysis of the different features. In the Cookie Theft Picture, these features can be summarized as follows^24^:

- The salience of information, with the action of the three characters (mother, son, and daughter) appearing more saliently than the background details (garden, items in the sink, etc.).
- Semantic categorization and referential cohesion: identification of the people, objects, and actions present in the scene and the use of pronouns to refer to them.
- Causal and temporal relations (e.g., between the mother not paying attention, the attempt to steal the cookies, and the imminent fall of the boy).
- Attribution of mental states (the mother is daydreaming, and the children are taking advantage of that).
- General cognition and perception of the whole scene.
- Structural language: phonology, syntax, and semantic aspects, as well as speech production.

Furthermore, discourse production requires the ability to create coherence, or conceptual connection (logical, causal, chronological, etc.), between the utterances at local and global levels. This goal is achieved at the local level by ensuring that consecutive sentences are presented cohesively in a continuous flow of information without abrupt shifts or interruptions. At the global level, the speaker must stick to the thematic thread and overarching plot. Achieving both coherence levels requires executive (planning) and working memory resources^25^.

Regarding the microlinguistic aspects (referring to syntax and phonology), there were no relevant differences between patients and controls. There was only a tendency of BD patients to produce shorter sentences with some paragrammatical errors, without statistical significance.

In the macrolinguistic aspects (referring to the textual organization and information content), BD patients produced more cohesion errors than controls, both in oral and written modalities. Textual cohesion concerns the appropriate use of grammatical articulation elements and connective terms, which produce a harmonious connection between sentences, periods, and paragraphs of a text. Therefore, it is a function related to the syntactic competence of the individual that might be associated with the syntactic processing difficulty already described in BD patients^11,20^. However, we did not find an association between the number of cohesion errors and performance in TROG-2.

Another finding of our study was a lower number of thematic units produced by the BD group in the oral modality. The production of thematic units from a picture is related to several stages of cognitive processing that begin with the correct visual perception of the stimulus and culminate in the integration and interpretation of the visualized content. However, we did not find any associations between the patients’ performance in the production of thematic units and tests of attention and nonverbal memory, which may be explained partially because the picture was available for visualization throughout the task. Moreover, in the written modality, the number of thematic units produced was similar to that of controls. Contrasting to our findings, Perlini et al.^11^ showed only reduced mean length of utterances in the BD group. However, it must be noted that Perlini’s study was conducted in a younger cohort and only in the oral modality.

Finally, when comparing the structure of texts produced by the two groups according to the modality (BD: oral vs. written; controls: oral vs. written), we found structural similarity regarding the greater number of words and elocutions, with consequent reduction of the percentage of lexical informativeness in the oral modality in both groups, which reflects inherent characteristics of these two forms of output (written output being better organized and more synthetic) and the absence of differences in most micro- and macrolinguistic elements (syntactic organization, lexical selection, local cohesion and coherence, and content). Across-modality differences were (taking the written narrative as reference) (1) the BD group produced longer and more complex sentences and (2) controls presented a lower number of global coherence errors. Such findings also reflect intrinsic characteristics of written texts and should not be regarded as indicative of intergroup differences in language processing.

In line with the findings described in clinical language studies, abnormalities in functional connectivity between temporal and frontal areas in euthymic BD patients compared to healthy controls have been demonstrated by electrophysiological^26^ and neuroimaging studies^27^.

This study showed that euthymic elderly patients with BD presented minimal changes in language use in a descriptive discourse task, both in the oral and written modalities. The mild macrolinguistic alterations found in this group (increase in the number of cohesion errors) may be related to the syntactic difficulties already described in BD^11,20^, although we could not demonstrate this association in our study. Regarding the lower number of thematic units produced by patients with BD in the oral modality (but not in the written modality), we hypothesize that such a finding may derive from attentional and executive mechanisms that would be more active in the written modality; however, once again, our data were not able to corroborate this hypothesis.

The main limitations of this study are its specificity toward a restricted group of BD patients (elderly people) and the possible influence of drug treatment on cognitive functions in the BD group. We believe that studies with a greater number of individuals and employing other forms of discourse (spontaneous, narrative, and procedural) combined with functional brain studies may complement our findings and increase our understanding of discourse language abilities in BD.

## References

[1] Ladeira RB, Nunes PV, Forlenza OV, Radanovic M, Aprahamian I Neuropsiquiatria geriátrica.

[2] Miskowiak KW, Burdick KE, Martinez-Aran A, Bonnin CM, Bowie CR, Carvalho AF (2018). Assessing and addressing cognitive impairment in bipolar disorder: the International Society for Bipolar Disorders Targeting Cognition Task Force recommendations for clinicians. Bipolar Disord.

[3] Szmulewicz A, Valerio MP, Martino DJ (2020). Longitudinal analysis of cognitive performances in recent-onset and late-life bipolar disorder: a systematic review and meta-analysis. Bipolar Disord.

[4] Forlenza OV, Aprahamian I, Radanovic M, Talib LL, Camargo MZ, Stella F (2016). Cognitive impairment in late-life bipolar disorder is not associated with Alzheimer’s disease pathological signature in the cerebrospinal fluid. Bipolar Disord.

[5] Kohler S, Marlinge E (2019). Cognitive aging of bipolar patients. Geriatr Psychol Neuropsychiatr Vieil.

[6] Weiner L, Doignon-Camus N, Bertschy G, Giersch A (2019). Thought and language disturbance in bipolar disorder quantified via process-oriented verbal fluency measures. Sci Rep.

[7] Bora E (2018). Neurocognitive features in clinical subgroups of bipolar disorder: a meta-analysis. J Affect Disord.

[8] Raucher-Chéné D, Achim AM, Kaladjian A, Besche-Richard C (2017). Verbal fluency in bipolar disorders: a systematic review and meta-analysis. J Affect Disord.

[9] Sung K, Gordon B, Vannorsdall TD, Ledoux K, Schretlen DJ (2013). Impaired retrieval of semantic information in bipolar disorder: a clustering analysis of category-fluency productions. J Abnorm Psychol.

[10] Curtis VA, Thompson JM, Seal ML, Monks PJ, Lloyd AJ, Harrison L (2007). The nature of abnormal language processing in euthymic bipolar I disorder: evidence for a relationship between task demand and prefrontal function. Bipolar Disord.

[11] Perlini C, Marini A, Garzitto M, Isola M, Cerruti S, Marinelli V (2012). Linguistic production and syntactic comprehension in schizophrenia and bipolar disorder. Acta Psychiatr Scand.

[12] World Health Organization (2004). ICD-10: international statistical classification of diseases and related health problems: 2^nd^ ed.

[13] Hamilton M (1960). A rating scale for depression. J Neurol Neurosurg Psychiatry.

[14] Young RC, Biggs JT, Ziegler VE, Meyer DA (1978). A rating scale for mania: reliability, validity and sensitivity. Br J Psychiatry.

[15] Smith GE, Ivnik RJ, Petersen RC (2003). Mild cognitive impairment.

[16] Goodglass H, Kaplan E (1972). The assessment of aphasia and related disorders.

[17] Bora E, Yücel M, Pantelis C (2010). Neurocognitive markers of psychosis in bipolar disorder: a meta-analytic study. J Affect Disord.

[18] Tréhout M, Leroux E, Delcroix N, Dollfus S (2017). Relationships between corpus callosum and language lateralization in patients with schizophrenia and bipolar disorders. Bipolar Disord.

[19] Radanovic M, Nunes PV, Gattaz WF, Forlenza OV (2008). Language impairment in euthymic, elderly patients with bipolar disorder but no dementia. Int Psychogeriatr.

[20] Radanovic M, Nunes PV, Forlenza OV, Ladeira RB, Gattaz WF (2013). Cognitive-linguistic deficits in euthymic elderly patients with bipolar disorder. J Affect Disord.

[21] Andreou C, Bozikas VP, Ramnalis A, Giannakou M, Fokas K (2013). Semantic priming in remitted patients with bipolar disorder. J Behav Ther Exp Psychiatry.

[22] Raucher-Chéné D, Terrien S, Gobin P, Gierski F, Caillies S, Kaladjian A (2019). Differential semantic processing in patients with schizophrenia versus bipolar disorder: an N400 study. Acta Neuropsychiatr.

[23] Lee CW, Kim SH, Shim M, Ryu V, Ha RY, Lee S (2016). P600 alteration of syntactic language processing in patients with bipolar mania: comparison to schizophrenic patients and healthy subjects. J Affect Disord.

[24] Cummings L (2019). Describing the cookie theft picture: sources of breakdown in Alzheimer’s dementia. Pragmatics and Society.

[25] Kemmerer D, Kemmerer D (2015). Cognitive neuroscience of language.

[26] Painold A, Faber PL, Reininghaus EZ, Mörkl S, Holl AK, Achermann P (2020). Reduced brain electric activity and functional connectivity in bipolar euthymia: An sLORETA source localization study. Clin EEG Neurosci.

[27] Reinke B, van de Ven V, Matura S, Linden DE, Oertel-Knöchel V (2013). Altered intrinsic functional connectivity in language-related brain regions in association with verbal memory performance in euthymic bipolar patients. Brain Sci.

